# Comparison of Intraocular Pressure Measurements in Healthy Pediatric Patients using Three Types of Tonometers

**DOI:** 10.4274/tjo.92593

**Published:** 2017-01-17

**Authors:** Muhsin Eraslan, Eren Çerman, Sena Sümmen

**Affiliations:** 1 Marmara University Faculty of Medicine, Department of Ophthalmology, İstanbul, Turkey

**Keywords:** Pediatric intraocular pressure, Tono-Pen tonometry, Goldmann applanation tonometry, non-contact tonometry, central corneal thickness

## Abstract

**Objectives:**

This study aimed to compare intraocular pressure (IOP) measurements in healthy pediatric patients using three types of tonometers.

**Materials and Methods:**

Seventy-eight eyes of 78 patients under the age of 18 who underwent a routine ophthalmologic examination were included in the study. IOP was measured using Tono-Pen (TP) tonometry, Goldmann applanation tonometry (GAT), and non-contact tonometry (NCT), consecutively. IOP was adjusted based on central corneal thickness (CCT). Patients with any ocular disorders other than a limited refractive error were excluded from the study.

**Results:**

The study consisted of 46 girls and 32 boys. The mean age was 12.6±2.7 (range: 5-17) years. The mean CCT was 559.3±35.3 µm. The mean refractive error was -0.50±1.70. The mean level of visual acuity was 0.98±0.1 (range: 0.3-1.0) using the Snellen chart. Significant differences were found between the measurement results of each of the three tonometric methods. Mean IOP was 12.1±2.2 mmHg for TP, 15.7±2.5 mmHg for GAT, and 17.1±3.1 mmHg for NCT. The correlations between measurement methods revealed that the highest correlation was between NCT and GAT (p<0.001, r=0.670). The second highest correlation was between NCT and TP (p<0.001, r=0.477). The lowest correlation was between GAT and TP (p<0.001, r=0.403). A positive correlation was found between CCT and each IOP measurement method.

**Conclusion:**

In pediatric patients, TP and NCT measurements were found to be positively correlated with GAT measurements. Because TP measurements were lower than GAT measurements and NCT measurements were higher than GAT measurements, patient follow-ups, treatment strategies, and surgery plans must be organized taking these differences into consideration.

## INTRODUCTION

Despite the important role of cornea and optic nerve appearance in the diagnosis of pediatric glaucoma, intraocular pressure (IOP) measurement is still the primary diagnostic method. In addition, IOP remains the only risk factor that can be altered in glaucoma therapy and these modifications have been proven to be able to prevent disease progression.^1^ This makes the accurate measurement of IOP particularly important. The evaluation of IOP in pediatric cases may vary depending on patient cooperation. Stress caused by devices which contact the cornea may cause the patient to cry, leading to the Valsava maneuver and increasing systemic venous pressure and IOP.^2^ It is therefore recommended to conduct the examination under general anesthesia in patients with suspected glaucoma. However, IOP measurements conducted in the outpatient clinic setting can are informative when deciding which patients to examine under general anesthesia.

Previous studies have demonstrated that measurements made using Schiotz tonometry may lead to inaccurate results due to factors such as corneal curvature incompatibility and corneal diameter.^3^ For this reason, the Goldmann applanation tonometer (GAT) is currently accepted as the gold standard tonometer. Unfortunately, measuring with the GAT is not possible with pediatric patients of all ages. Although some studies have demonstrated good agreement between measurements made with the Tono-Pen (TP), GAT and non-contact tonometer (NCT),^4,5^ in clinical practice these instruments can yield very different results. This can have an impact on treatment decisions and in some cases even surgery decisions. Because IOP assessment may lead to legal issues in certain situations, it is critical to evaluate measurement reliability and determine the factors affecting measurement.

The aim of this study was to compare IOP measurements made in outpatient clinic conditions with TP, GAT and NCT in pediatric patients amenable to IOP measurement in a sitting position.

## MATERIALS AND METHODS

Seventy-eight eyes of 78 patients examined in our ophthalmology clinic between April and June 2015 were included in the study. Only the patients’ right eyes were included. Patients had no ocular disease other than refractive errors. Exclusion criteria included: hypermetropia or myopia greater than 4 diopters (D); corneal astigmatism greater than 2.5 D; any known ocular disease or suspicion of glaucoma (history of high IOP, deep or large optic pit, family history, etc.); history of ocular surgery; periocular steroid use during or within 3 months prior to the study; use of any systemic or ophthalmic drugs which may affect IOP; and inability to comply with any of the assessment methods utilized in the study. The study was conducted in accordance with the Declaration of Helsinki and was approved by the local clinical research ethics committee. Informed consent was obtained from the patients’ legal guardians before each procedure.

IOP measurements were conducted using the TP (Tono-Pen Avia, Reichert, USA), GAT (Haag-Streit, Switzerland) and NCT (Nidek NT 530, Japan). The same tonometers were used throughout the study. As recommended by the manufacturers, the TP and GAT were calibrated daily and the NCT was calibrated once a month. Measurements were performed by the same physician before dilating the pupil, instilling a topical anesthetic (Alcaine proparacaine hydrochloride; Alcon, Fort Worth, Texas, USA), and fluorescein stain (Norvatis fluorescein; Norvatis, Basel, Switzerland) for GAT. Measurements were taken at 5-minute intervals, and the average of 3 measurements was taken for each device. All measurements were done with the patient in a seated position. Measurements were taken with the three instruments in the following order: TP, GAT, NCT. This was followed by central corneal thickness (CCT) measurement using the Pachymeter SP-3000 (Tomey, USA) pachymetry instrument, then a corrected IOP value was calculated based on the CCT value: corrected IOP=Measured IOP-(CCT-545)/50x2.5 mmHg.^6^

SPSS version 17.0 (SPSS Inc., Chicago, IL, USA) software package was used for statistical analyses. P values less than 0.05 were accepted as statistically significant. Independent samples t-test was used for intergroup comparisons. Pearson’s test was used to determine the presence of correlations. Differences of 1.96 standard deviations from the mean were used when calculating the limits of agreement. Associations between differences and means were analyzed using Bland-Altman plots.

## RESULTS

A total of 78 subjects were included in the study; 18 were later excluded due to noncompliance with at least 1 of the measurement techniques.

Of the subjects included in the study, 46 were female and 32 were male. The mean age was 12.6±2.7 (range, 5-17) years. Mean CCT was 559.3±35.3 µm and mean refraction value was -0.50±1.70 D. Visual acuity on Snellen chart was 0.98±0.1 (range, 0.3-1.0).

**Figure 1:**
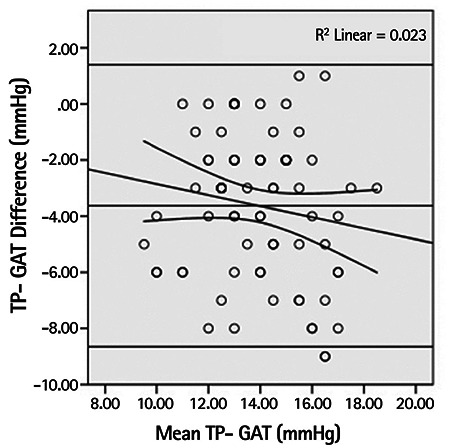
Bland-Altman scatter plot showing the errors in intraocular pressure measurements obtained with the Tono-Pen compared to Goldmann applanation tonometry results TP: Tono-Pen, GAT: Goldmann applanation tonometry

**Figure 2:**
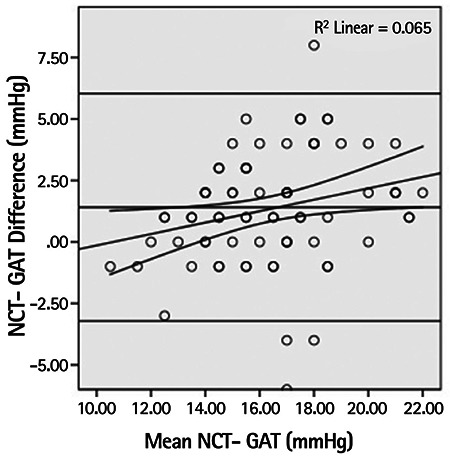
Bland-Altman scatter plot showing the errors in intraocular pressure measurements obtained with non-contact tonometer compared to Goldmann applanation tonometry results NCT: Non-contact tonometer, GAT: Goldmann applanation tonometry

Mean IOP was measured as 12.1±2.2 mmHg with TP, 15.7±2.5 mmHg with GAT and 17.1±3.1 mmHg with NCT. The differences and 95% confidence intervals between these mean values are shown in the Bland-Altman plots in Figures 1 and 2.

CCT positively correlated with measured IOP measurements obtained from all of the devices (TP: r=0.305, p=0.007; GAT: r=0.355, p=<0.001; NCT: r=0.471, p<0.001).

A weak negative correlation emerged between age and the difference between NCT and GAT values (r=-0.225, p=0.048), while there was no significant relationship between age and the difference between TP and GAT values (r=0.126, p=0.271).

Moderate correlations were observed between all of the measurement methods (TP-GAT: r=0.403, p<0.001; NCT-GAT: r=0.670, p<0.001; NCT-TP: r=0.477, p<0.001).

CCT values were not significantly correlated with the amount of deviation of TP and NCT measurements from GAT measurements (p>0.05).

**Figure 3:**
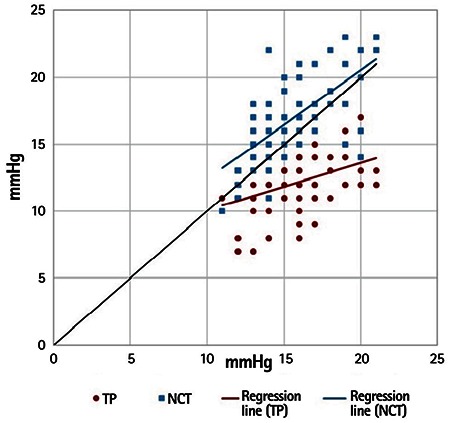
Correlation between the results of Goldmann applanation tonometry and measurements obtained using the Tono-Pen and non-contact tonometer. A moderately significant correlation was found between the measurements TP: Tono-Pen, NCT: Non-contact tonometer

There was a moderate positive correlation between the TP-GAT difference and significantly rising mean IOP values (r=0.459, p<0.001), whereas a weak negative correlation was observed between the NCT-GAT difference and significantly rising mean IOP values (r=-0.260, p=0.021) (Figure 3).

The Cronbach alpha reliability coefficient of the measurements made with the 3 different instruments was 0.762. The reliability coefficient between TP and GAT was 0.571, indicating low reliability; the Cronbach’s alpha of 0.793 between NCT and GAT showed sufficiently reliable agreement.

## DISCUSSION

Fewer comparative studies have been performed in pediatric patients than in adults. Although the TP may be easier to use with younger patients than the GAT and NCT, the results of our study show that measuring with the TP may yield IOP values which are artificially low. In this study we determined that NCT and GAT measurements show adequate reliability, while TP measurements showed low reliability. Furthermore, the difference between TP and GAT measurements grew as IOP values increased. This finding is consistent with previous studies demonstrating that the TP measures significantly lower than the GAT at IOP values over 20 mmHg.^7^

Other studies have shown that the NCT yields higher values than the GAT and that this difference increases as CCT increases.^8^ In the present study, the discrepancies between GAT measurements and those made with TP and NCT were uncorrelated with CCT but were associated with IOP elevation. In their 2006 publication, Alagöz et al.^9^ reported that the NCT gave significantly higher IOP values compared to the GAT, even in patients with normal IOP values. Akman et al.^10^ obtained similar results using the NCT and GAT in subjects with normal IOP and recommended using the NCT as a screening test and confirming high values with the GAT. In a similar study from 2002, Güler et al.^11^ found good agreement between the NCT and GAT and concluded that the NCT was a convenient, reproducible and reliable method.

Consistent with the results of the present study, Feng et al.^12^ reported that the NCT yielded slightly higher values than the GAT in their 2015 study including 419 pediatric patients. They emphasized that the NCT may be a preferable method because it does not require local anesthetic.

In contrast, Buscemi et al.^13^ conducted a study with 42 pediatric patients and argued that, compared to the GAT, the NCT may yield false negative results in pediatric patients under 9 years old and should not be used with patients under this age.

A previous study demonstrated that differences due to postural changes resulted in low reliability between measurements taken with the TP and pneumatic tonometry and those taken with the GAT.^14^ As all measurements were taken with patients in a seated position, any differences arising due to postural changes are not an issue in the present study. However, Takenaka et al.^15^ determined that the reliability of IOP measurements may be lower in children who move during assessment. Therefore, only cooperative subjects who were able to remain motionless during measurement were included in the present study. Despite this, the difference between GAT and NCT measurements significantly decreased with older age, while TP measurements were not affected. Furthermore, because TP measurement was done first, it is possible that the subjects may not have been able to sufficiently cooperate.

Many earlier studies have demonstrated the link between CCT and measured IOP values.^16,17^ As none of the tonometers used in the present study allowed the evaluation of CCT or other parameters which may affect IOP measurement such as ocular rigidity or hysteresis, we used IOP values adjusted for CCT in our analysis. The formula we used in the present study was developed in 2004 by Shih et al.^6^ based on a mathematical formula proposed by Orssengo and Pye18 in their in vivo human cornea studies investigating the association between corneal elasticity and accurate IOP measurement.

### Study Limitations

One of the limitations of our study was the order of the devices used for measurements. The use of two different contact tonometers made it impossible to prevent possible IOP changes due to corneal compression or aqueous massage. However, opposite to the expected outcome, measurement with the NCT after both contact tonometers yielded the highest IOP values, while the first measurement using the TP was lowest. It is therefore unlikely that the order we used impacted the results, but there is a slight possibility that it caused the underestimation of discrepancies which may have been more pronounced using a different measuring order.

Another limitation of our study was that the mean age of the subjects was 12.6±2.7 years. Larger studies which also include younger subsets of the pediatric population are needed.

## CONCLUSION

Measurements obtained with both the TP and NCT were positively correlated with those from the GAT, the accepted gold standard IOP assessment method. It is expected that TP values will be lower and NCT values will be higher than GAT values, and these differences should be considered when following patients and making decisions regarding treatment and surgery. This study also demonstrated that measurements obtained with the TP are less reliable compared to those from the NCT, and that this discrepancy may be more pronounced at high IOP levels.

### Ethics

Ethics Committee Approval: Available, Informed Consent: Available.

Peer-review: Externally peer-reviewed.
